# Development of a Machine-Learning Intrusion Detection System and Testing of Its Performance Using a Generative Adversarial Network

**DOI:** 10.3390/s23031315

**Published:** 2023-01-24

**Authors:** Andrei-Grigore Mari, Daniel Zinca, Virgil Dobrota

**Affiliations:** Communications Department, Technical University of Cluj-Napoca, 400114 Cluj-Napoca, Romania

**Keywords:** generative adversarial network, intrusion detection system, intrusion evasion, machine learning, NSL-KDD dataset, Python

## Abstract

Intrusion detection and prevention are two of the most important issues to solve in network security infrastructure. Intrusion detection systems (IDSs) protect networks by using patterns to detect malicious traffic. As attackers have tried to dissimulate traffic in order to evade the rules applied, several machine learning-based IDSs have been developed. In this study, we focused on one such model involving several algorithms and used the NSL-KDD dataset as a benchmark to train and evaluate its performance. We demonstrate a way to create adversarial instances of network traffic that can be used to evade detection by a machine learning-based IDS. Moreover, this traffic can be used for training in order to improve performance in the case of new attacks. Thus, a generative adversarial network (GAN)—i.e., an architecture based on a deep-learning algorithm capable of creating generative models—was implemented. Furthermore, we tested the IDS performance using the generated adversarial traffic. The results showed that, even in the case of the GAN-generated traffic (which could successfully evade IDS detection), by using the adversarial traffic in the testing process, we could improve the machine learning-based IDS performance.

## 1. Introduction

Network security is one of the fastest growing industries due to the increasing number and sophistication of attacks. Data are considered to be among the most valuable assets of a company. Cyber-attacks are usually launched against a user’s network and consist of exploiting a vulnerability to enable unauthorized use of electronic data. Current networks encounter proliferating and increasingly sophisticated attacks. Therefore, network protection against cyber-attacks is of utmost importance. Intrusion detection systems (IDSs) are deployed in defense-in-depth scenarios in order to protect networks from various types of attacks. Depending on the placement of the device and whether it tries to prevent attacks, the system can act also as an intrusion prevention system (IPS). In general, an IDS monitors network traffic to search for signatures of malicious activity or violations of rules created based on security policies. Signature-based and anomaly-based methods are the two main approaches used to build such systems. A signature represents an indicator of compromise created based on previously identified (known) threats. In the case of unknown threats or zero-day attacks, anomaly-based detection systems are more appropriate. The drawbacks of anomaly-based IDSs include higher rates of false positives (classifying normal traffic as attacks) and false negatives (allowing malicious traffic to propagate). According to [1], various techniques can be used in order to build an anomaly-based detection system: threshold detection, either static or heuristic; statistical measures; rule-based measures; and machine learning (ML).

### 1.1. Objectives

Although signature-based IDSs are more common, recent developments in anomaly-based IDSs use machine-learning algorithms. A model uses one or more algorithms to learn to recognize malicious activity. Each model is built using a certain set of features that are available for a specific dataset. The accuracy of machine learning IDSs with test datasets can be higher than 90%, as demonstrated in Section 2.2. Unfortunately, an attacker can evade detection by machine learning IDSs by generating malicious traffic that retains the functional features of attack traffic. In Section 2.3, we present a GAN architecture that can be used in order to test and improve IDS performance in the presence of adversarial traffic.

### 1.2. Dataset Used

ML algorithms [2,3,4,5,6] are currently being used to successfully develop IDSs. Note that the quality and the relevance of the training dataset [7] (e.g., NSL-KDD, UNSWNB15) are of great importance for the final accuracy. A review of the available datasets that have been used for machine-learning applications is presented in [8].

For the purposes of this paper, we used the NSL-KDD dataset, which is a refined version of its predecessor KDD’99, a well-known benchmark in the research on intrusion detection techniques [6,9,10,11,12,13]. This labeled dataset is split into training and testing files and can be downloaded from [12]. In addition to the normal traffic, there are 23 attack types documented (for example, neptune, ipsweep, portsweep), which are grouped into four main categories: denial of service (DoS), probing (probe), user to root (U2R), and remote to user (R2L) [6]. The DoS category refers to attacks that consume the server’s available resources, making it difficult for the attacked system to fulfill legitimate user requests. A probing attack usually precedes an access attack and consists of collecting information about the victim, usually by scanning IP addresses and ports. A user to root attack attempts to obtain unauthorized root access in a system where the attacker already has user access. In a remote to user case, an unauthorized connection from a remote system under the attacker’s control is attempted in order to gain local access. For each network traffic record, there are 41 types of features, and these are classified as attack or normal types; 9 of them are discrete values and the remaining 32 values are continuous. Discrete data employ values that can only take on specific values and cannot be subdivided. Continuous data employ values that can take on any numerical value and can be subdivided into smaller parts. These features can be classified into four categories: basic, content, time-related traffic, and host-based traffic [6,13].

### 1.3. Machine-Learning Algorithms Implemented

Several machine-learning algorithms were used in this study to implement the IDS.

*K-Nearest Neighbors*

The K-nearest neighbors (KNN) algorithm is a data classification method for estimating the likelihood that a data point will become a member of one group or another based on which groups the data points nearest to it belong to. This non-parametric algorithm is used in the case of classification problems. We implemented the K-nearest neighbors algorithm by using the KNeighborsClassifier class from the sklearn library.

*Decision Tree*

Decision trees are supervised learning techniques that can be used in the case of classification problems. They are tree-structured classifiers, where internal nodes represent the features of a dataset, branches represent the decision rules, and each leaf node represents an outcome. The decisions are made on the basis of the features of the given dataset.

We implemented the decision tree by using the DecisionTreeClassifier class from the sklearn library.

*Random Forest*

The random forest algorithm is a supervised machine-learning algorithm that is used widely in classification and regression problems. It builds decision trees with different samples and calculates the majority class for classification and average prediction for regression. We implemented the random forest algorithm by using the RandomForestClassifier class from the sklearn library.

*Support Vector Machine*

A support vector machine (SVM) is a very powerful and versatile machine-learning model capable of performing linear or nonlinear classification, regression, and outlier detection. We implemented it using the SVC class from the sklearn library.

*Neural Networks*

Neural networks, also referred to as artificial neural networks (ANNs) or simulated neural networks (SNNs), are a subgroup of machine-learning models and are at the core of deep-learning algorithms. Even though they are a subset of machine-learning models, neural networks are handled separately as they had the greatest impact in the implementation we realized.

We implemented an artificial neural network using the Keras API with Relu and LeakyRelu as the activation functions.

*Generative Adversarial Networks*

Generative adversarial networks (GANs) represent a recent development in machine learning and are a powerful class of neural networks that are utilized in unsupervised learning. They are generative models in the sense that they create new data instances that resemble the original training data. New data are created based on learning of the patterns in the original data. Some of the first examples used images as training data. The initial paper on GANs [14] described their components; for instance, GANs can be understood as two participants in a game where each player tries to minimize their cost based on the parameters they control and the other player’s output. In this context, each player will reach a point called the local Nash equilibrium (from game theory) with the minimum cost, which can be computed based on the parameters. There are two players, the generator and the discriminator. The generator can take an input from a source of randomness (for example, a uniform distribution) and should be able to generate outputs that are similar to the training data used by the other player. These outputs are useful when the local equilibrium is reached. The second player is the discriminator and uses inputs from the original training set and from the output of the generator. The discriminator predicts the probability that the input from the generator is real or fake. The discriminator cost is minimized when it correctly classifies the original and the fake data. On the other hand, the generator cost is minimized when it successfully generates adversarial data that are incorrectly classified as real.

**Figure 1:**
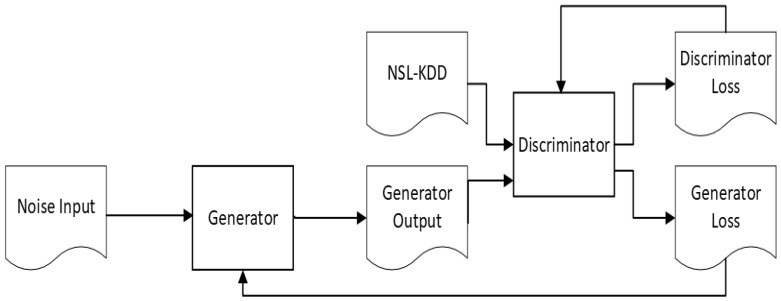
Generative adversarial network.

There are several applications of GANs in network security. One approach is to use the output from the generator as test data and to consider a black-box IDS as a discriminator; its performance is then tested against adversarial data. In [13], a framework called IDSGAN is described in which a generator transforms original malicious traffic into adversarial traffic examples that are later sent to an IDS. The framework proposed inspired us to create and test an alternative solution, which is detailed in this paper. This solution not only makes it possible to assess the performance of various IDS implementations against adversarial traffic but also, more important, allows for the improvement of IDS detection by including generated adversarial traffic in the training phase of the IDS. Other research papers are discussing different approaches to use GAN de detect network intrusions [15,16,17,18,19,20,21]. The principle of the GAN is shown in Figure 1.

## 2. Materials and Methods

This section presents the platforms and components used for the development environment, followed by a step-by step description of the development of the machine-learning IDS and the process of testing and tuning the performance of the IDS using a GAN.

### 2.1. Platforms and Components Used for the Development Environment

#### 2.1.1. TensorFlow

TensorFlow is one of the most powerful platforms for creating and maintaining deep-learning applications. It was launched by Google as an end-to-end machine-learning platform [22], and it was initially used for projects related to areas such as recommendation engines and even translation. It is an open-source platform created for the automation of machine-learning applications. It contains tools, libraries, and resources that allow developers to build and deploy machine-learning applications for the entire product lifecycle.

TensorFlow can run on both graphics processing units (GPUs) and central processing units (CPUs). Some components use NVidia’s CUDA language to increase performance in environments where GPUs are used. However, most of the time, TensorFlow applications are developed on desktops with CPUs only. The training of the model takes place on these devices and, after it is completed, the model can be deployed on the cloud, on various desktop platforms running current operating systems, or even on mobile devices or IoT devices.

#### 2.1.2. Keras API

Keras is a high-level neural network API written in Python that uses machine-learning computation platforms as its backend, such as TensorFlow (the default one), CNTK, or Theano [23]. TensorFlow adopted Keras as the default high-level API in Python for its 2.0 release (the tf.keras package). It allows for fast prototyping, supporting both convolutional and recurrent networks (and combinations of the two), and can be used in both CPU and GPU environments.

To perform low-level operations, Keras must use a backend engine, the default being TensorFlow, as a mutual support between them. The backend can be changed by modifying a configuration file. Simpler applications can use the Sequential class model available in Keras. For more complex applications requiring a different arrangement of layers, the Model class with the functional API is available in Keras. In the case of the Model class, the layers are defined and then used to create the model. The learning and predicting processes are the same for both model types. Keras can be deployed across a vast range of platforms, including Google Cloud (by using the TensorFlow Serving library).

#### 2.1.3. Development Environment

Google Colab was chosen as the development environment because of its simplicity, ease of use, and the fact that it provides computational resources when the code is run on a virtual machine. In addition, it supports most of the machine-learning libraries available on the market. The environment provides most of needed libraries, such as numpy, pandas, tensorflow, sklearn, keras, etc., and it can be checked by running the command !pip freeze. Additional libraries can be installed by using the command !pip install.

**Figure 2:**
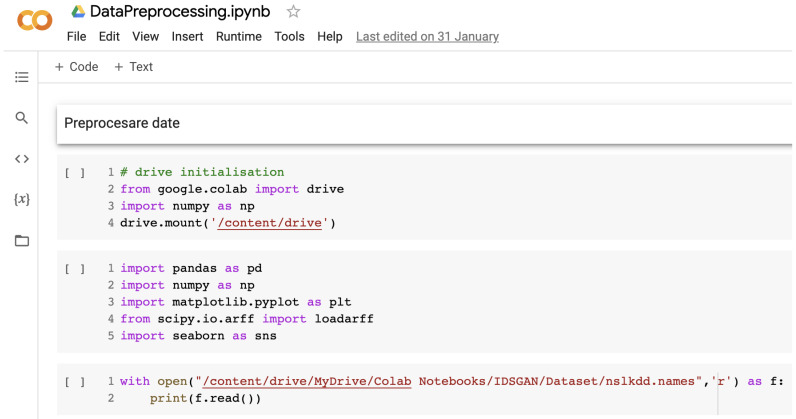
Screen capture of the Google Colab interface.

In order to maintain version control over the entire project, the GitHub platform was used. In order to synchronize our local repository with the remote one, we used the Google Colab “Save a copy in Git option”, which allowed us to actively commit to an existing repository. A screen capture of the Google Colab interface is presented in Figure 2.

### 2.2. Development of a Machine-Learning IDS

**Figure 3:**
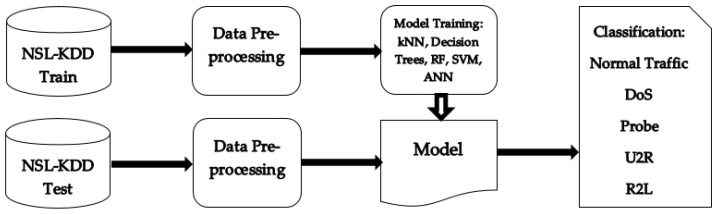
Proposed flow for the machine-learning IDS.

The steps involved in the development were data preprocessing, creation of the model, training and testing of the IDS, creation of the model, and then training of the IDS and testing of its performance using the GAN architecture. Figure 3 presents the proposed flow for the IDS using the machine-learning algorithms described in the previous section. The building blocks are described in Section 2.2.

In order to train the model, the NSL-KDD Train supervised dataset was used. The NSL-KDD Test dataset was used to test the model and evaluate its performance. These datasets are described in Section 1.2. The data preprocessing step is described in Section 2.2.1. The model was trained one algorithm at a time, and the entire process is described in Section 2.2.2. Then, in the testing phase, the model received inputs processed from the NSL-KDD Test dataset. The output of the model was a classification in five classes: normal traffic, DoS, probe, U2R, and R2L.

#### 2.2.1. Data Preprocessing

**Figure 4:**
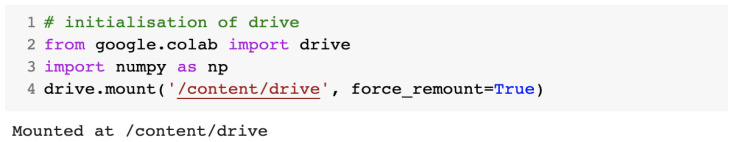
Mounting the drive to access the dataset.

The first implementation step in the development of the machine-learning IDS was data preprocessing. This step involves the improvement of the dataset quality, which will affect the IDS performance. The data preprocessing step included dataset splitting and loading, correlation, removal of the highly correlated features, and data entry normalization. The dataset was split into two files, the training dataset kdd_train.csv and the testing dataset kdd_test.csv. In addition to the NSL-KDD dataset, the additional metadata files nslkdd.names and training_attack_types were added, which provided information about the attack types in the dataset. These files were kept in the Google Drive directory that was created for the development of the system presented in this paper. In order to access these files, it was necessary to grant access to the Google Drive instance where the files were saved. After access was granted, the files could be loaded using the mount command. Figure 4 shows the commands that can be used to perform the mounting task.

**Figure 5:**
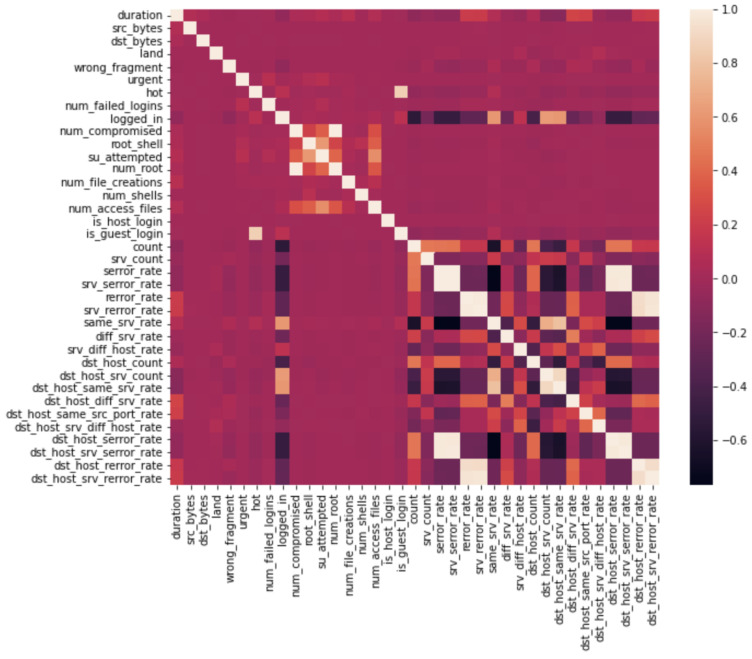
Heat map of the correlated features.

At runtime, authorization was requested to allow notebook access to the Drive location. The content of the file was loaded using the DataFrame property of the pandas library. Then, we mapped the attack entries to match the major attack categories probing (probe), denial of service (DoS), user to root (U2R), and remote to local (R2L). In the NSL-KDD files, the attack type was specified for each record. Based on the training_attack_types file, we performed the conversion to normal, DoS, probe, U2R, and R2L. Next, the data correlation step was performed. Correlation is a statistical measure that expresses the linear relationship between two variables. For this, we used the corr() method of the dataframe object. It is recommended that highly correlated features be removed from a dataset in order to improve the performance of machine-learning algorithms. Features with a high correlation have a value close to 1.0. For example, the features dst_host_srv_rerror_rate and srv_rerror_rate have a correlation value of 0.97. The heat map of the correlated features is presented in Figure 5.

The highly correlated features were removed from the dataset in order to improve the performance of the machine-learning algorithm. Once this step was completed, we were left with four categorical features (protocol_type, flag, service, and attack_type) that had to be mapped to numerical values. The service feature was dropped as it did not influence the learning process. We did not notice any performance improvement when the service parameter was present in the training and testing datasets. An alternative way to reduce the features is to use a BAT evolutionary algorithm [10].

**Figure 6:**
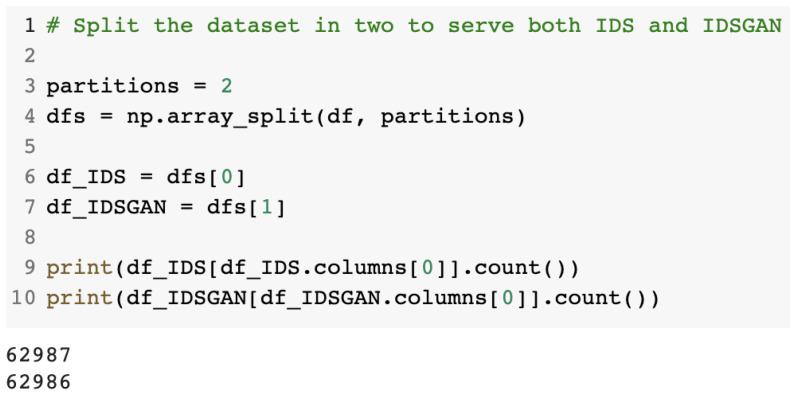
Dataset split in two to train the IDS and GAN models.

Next, the dataset was split in two to prevent overfitting of the models. The first half was used in the IDS model training, while the second was used for the GAN model training. Figure 6 presents the modality used to split the dataset in two: the first half for the IDS model training and the second for the GAN model training.

The training and testing of the GAN was undertaken using data from one attack type at a time. Therefore, the training data had to be further split into dedicated attack-type data frames.

**Figure 7:**
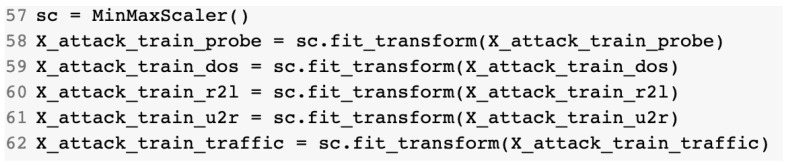
Training dataset normalization using MinMax.

The last step in the data preprocessing step was the normalization process for the data entries. The reason for the normalization was the fact that variables that are measured at different scales do not contribute equally to model training and are prone to create bias. In order to deal with this potential problem, feature-wise normalization, such as MinMaxScaling, was used before fitting the model. By doing so, all features were transformed into the [0, 1] range. Figure 7 presents the source code that used to apply MinMax normalization to the training dataset.

**Figure 8:**
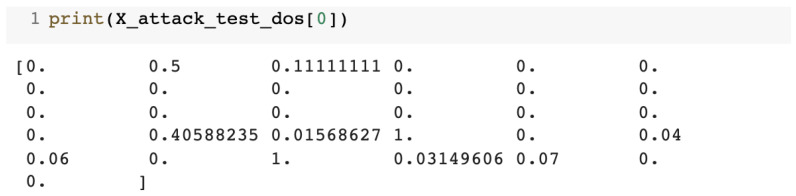
Record from the testing dataset for the DoS attack.

At the end of the preprocessing step, the dataset contained 31 normalized numerical features separated into dedicated attack-type training and test datasets. Figure 8 presents the test entry for one of the attacks (the DoS attack).

#### 2.2.2. Implementation of the machine-learning IDS

The IDS model creation step was realized in the IDS.ipynb notebook based on both classical machine-learning algorithms and neural network models. The first step consisted of loading the preprocessed dataset from the previous step. Once the dataset was loaded, we started training our IDS model. Five different algorithms were used:

- K-nearest neighbors (using the KNeighborsClassifier class from the sklearn library);
- Decision tree (using the DecisionTreeClassifier class from the sklearn library);
- Random forest (using the RandomForestClassifier class from the sklearn library);
- Support vector machine (using the SVC class from the sklearn library);
- Artificial neural network (using the Keras API with Relu and LeakyRelu as the activation functions).

**Figure 9:**
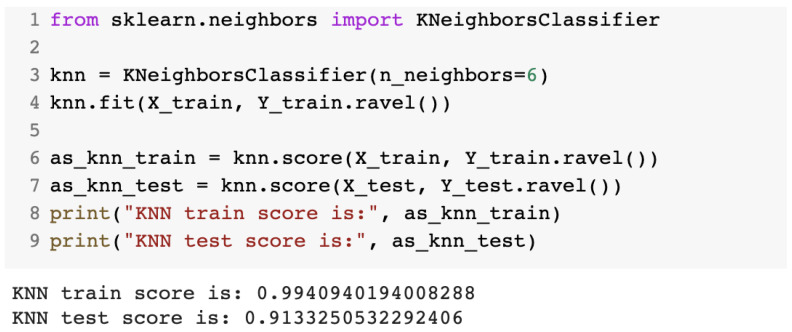
Source code for the IDS with the K-nearest neighbors algorithm.

The first algorithm used was K-nearest neighbors. From the sklearn library, we imported the KNeighborsClassifier class. The optimal K value primarily chosen was the square root of N, where N was the total number of data points. Figure 9 presents the source code for the K-nearest neighbors algorithm and the performance for the training and testing dataset.

**Figure 10:**
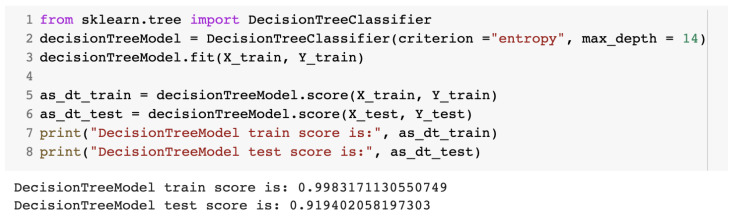
Source code for the IDS with the decision tree algorithm.

For the decision tree algorithm, the DecisionTreeClassifier class was imported from the sklearn library. The max_depth value represented the height of the binary tree. The value for max_depth was selected after numerous trials. Figure 10 presents part of the source code for the decision tree algorithm.

**Figure 11:**
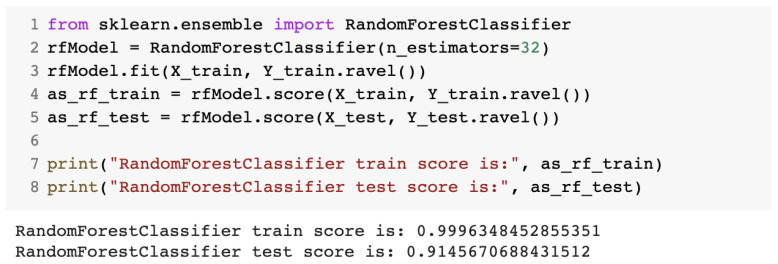
Source code for the IDS with the random forest algorithm.

The third algorithm was the random forest algorithm, for which we used the RandomForestClassifier class imported from the sklearn library. The number of estimators was selected after numerous trials. Figure 11 presents the source code for the random forest algorithm.

**Figure 12:**
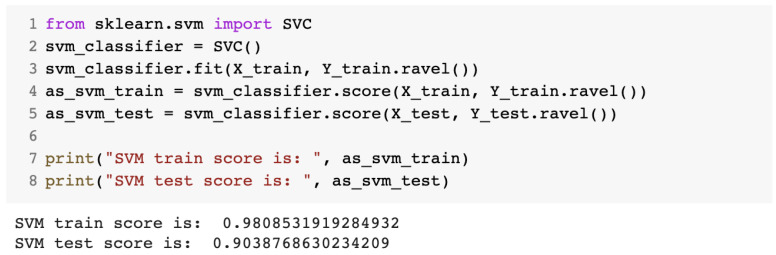
Source code for the IDS with the SVM algorithm.

The fourth algorithm was the support vector machine algorithm, for which we used the SVC class from the sklearn library. The source code for the IDS obtained with this algorithm is presented in Figure 12.

**Figure 13:**
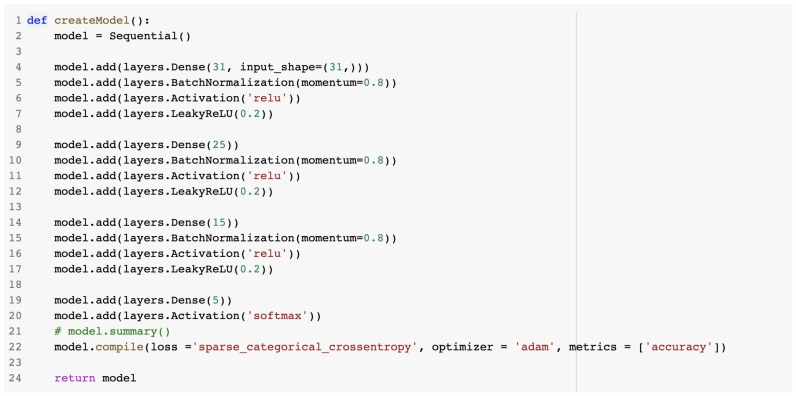
createModel method for the ANN IDS.

For the last model, the artificial neural network, the Keras API was involved. The model used the Keras Sequential class, which was a convenient way of adding a linear stack of layers. The architecture of the model was designed in such a way as to limit the number of hidden layers. The activation functions for the input layer and the hidden layers were the following: Relu and LeakyRelu. At the output layer, the Softmax activation function was chosen, predicting a multinomial probability distribution. Figure 13 presents the source code for the artificial neural network model.

**Figure 14:**
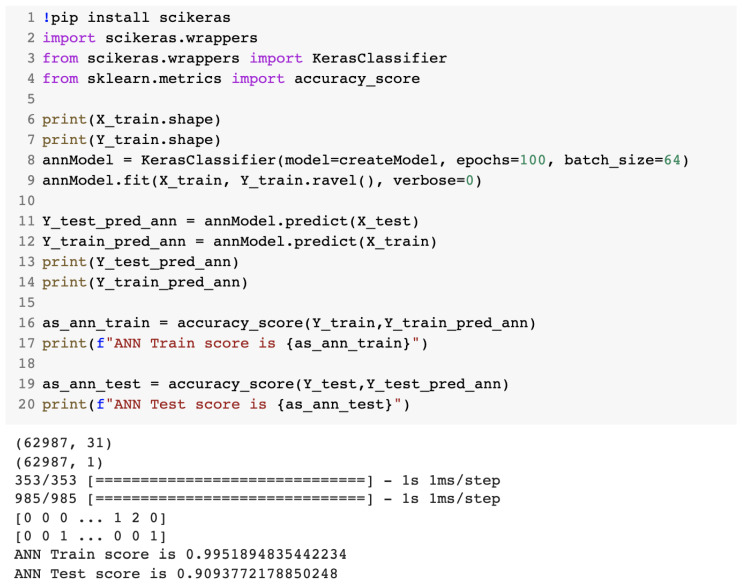
Source code for the training of the ANN IDS.

Next, we used the KerasClassifier class from the scikeras library, which provided us with a wrapper for the deep-learning models to be used for classification. Figure 14 presents the source code for the training of the ANN model.

#### 2.2.3. Performance Analysis of the Machine-Learning IDS

In the last step, all the IDS models were compared, and the first three classifiers were saved for use in the performance evaluation and training of the GAN. The models all performed very well when using the training dataset, exhibiting accuracy close to or over 0.9. However, only the ANN, random forest, and K-nearest neighbor classifiers were used in the next step. For the performance analysis [24], we computed the precision (or detection rate), recall, and F1 score (which combines both precision and recall) using Equations (1)–(3). In addition, we also show the confusion matrix.

(1) $$\mathrm{Precision}=\frac{TP}{TP+FP}$$

(2) $$\mathrm{Recall}=\frac{TP}{TP+FN}$$

(3) $$F1=2*\frac{Precision*Recall}{Precision+Recall}$$

where *TP* (true positive) represents the number of records correctly matched as attack traffic, *TN* (true negative) represents the number of records correctly matched as normal traffic, *FP* (false positive) represents the number of normal records incorrectly labeled as attack traffic, and *FN* (false negative) represents the number of attack records incorrectly labeled as normal traffic.

Table 1 presents the performance analysis and Table 2 presents the confusion matrix for the kNN IDS.

Table 3 presents the performance analysis and Table 4 presents the confusion matrix for the decision tree IDS.

Table 5 presents the performance analysis and Table 6 presents the confusion matrix for the random forest IDS.

Table 7 presents the performance analysis and Table 8 presents the confusion matrix for the SVM IDS.

Table 9 presents the performance analysis and Table 10 presents the confusion matrix for the ANN IDS.

The values in Table 1, Table 2, Table 3, Table 4, Table 5, Table 6, Table 7, Table 8, Table 9 and Table 10 were computed with the testing dataset. It was found that the machine-learning IDSs performed very well, especially for the normal, DoS, and probe classes with high numbers of records in the dataset. The U2R attack had a very low number of records in the training dataset and, with one exception (random forest), was not recognized correctly by the algorithms, raising a high number of false negatives. The high value for the precision parameter in the case of U2R was not a good indicator, as the total number of false positives was 0 and it did not take into consideration the high number of false negatives. Another point is that the usage of artificial neural networks seems promising, since even with a simple ANN the results were similar to the other algorithms tested. Compared with the results presented in [9], we obtained better results with the testing dataset for classes that were better represented in terms of the number of samples in the dataset. However, the authors of [9] obtained better performance using PPGO, a bio-inspired optimization technique, for the U2R class, which had few samples in the dataset.

### 2.3. Development of a GAN for Testing and Tuning of the IDS

In order to develop the GAN, the generator and the discriminator had to be implemented. To make testing easier, a configuration file was created. Then, the IDS performance had to be tested (using the models that were developed in the previous step).

#### 2.3.1. The Configuration File

**Figure 15:**
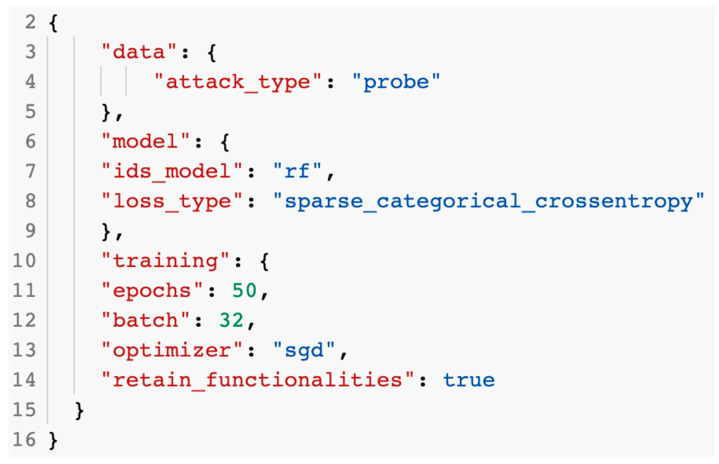
Contents of the configuration file.

To change the model’s parameters when testing the GAN against the IDS, a configuration file was created in the JSON format. Figure 15 presents the contents on the configuration file with the model’s parameters.

**Figure 16:**
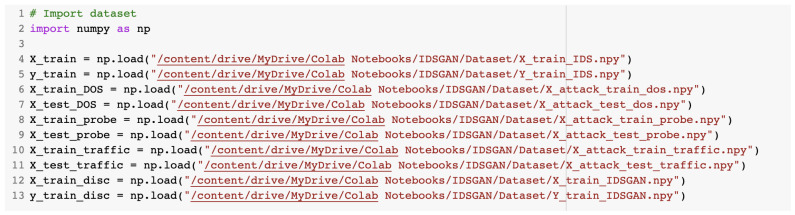
Loading of the dataset used for the GAN.

The configuration file data had to be loaded before being used for training or testing purposes. The creation and training of the GAN was undertaken in the IDSGAN.ipynb notebook. The dataset dedicated to the GAN was loaded from the Google Drive directory. The commands used to load the content of the dataset are presented in Figure 16.

**Figure 17:**
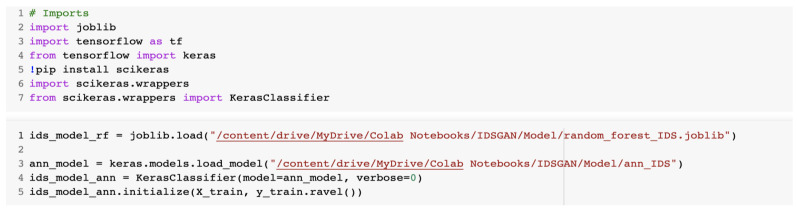
Loading of the models used for the GAN.

After that, we loaded the IDS models that were created in Section 2.2. Figure 17 presents the source code for loading of the models used for GAN.

**Figure 18:**
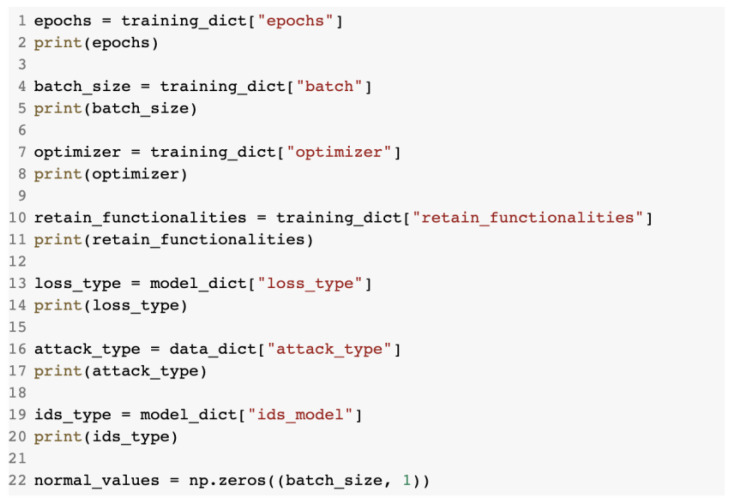
Loading the parameters from the configuration file.

The parameters of the model were loaded from the configuration file as is presented in Figure 18.

**Figure 19:**
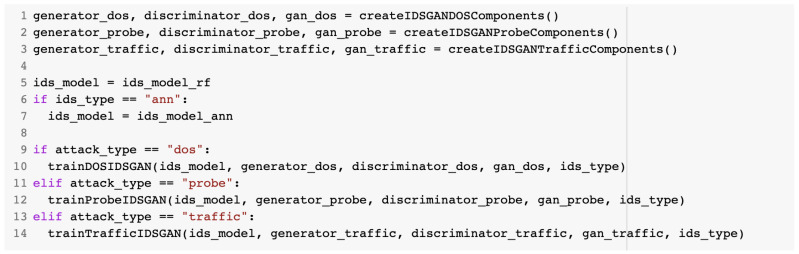
Training of the GAN according to the configuration file.

The training of the model was undertaken according to the configuration file data as is presented in Figure 19.

**Figure 20:**
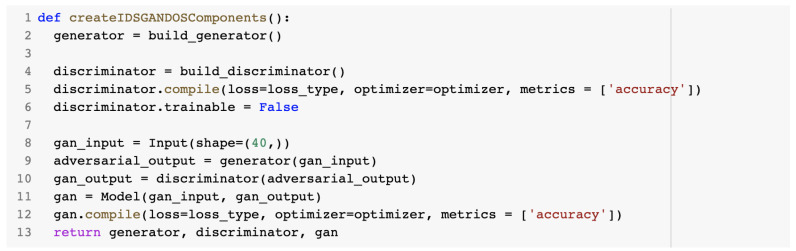
createIDSGANDOSComponents() method example for the DoS attack type.

The training of the model was undertaken according to the configuration file data. First, the components of the IDSGAN architecture were initiated. This was undertaken with the help of the createIDSGAN<attack_type>Components() method, which returned object instances of the GAN model, generator, and discriminator as is described in Figure 20.

This method involved the build_generator()and build_discriminator() methods, which created and returned the model instances for the generator and discriminator.

#### 2.3.2. The Generator Component of the GAN

**Figure 21:**
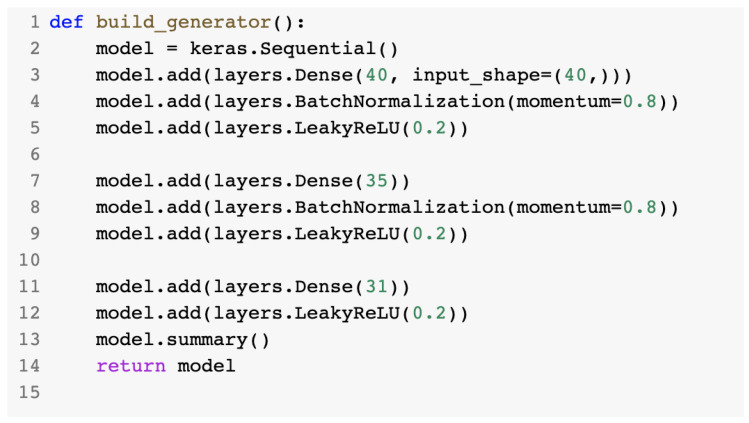
build_generator() method.

The build_generator() method created a sequential model with three layers. Its content is presented in Figure 21.

**Figure 22:**
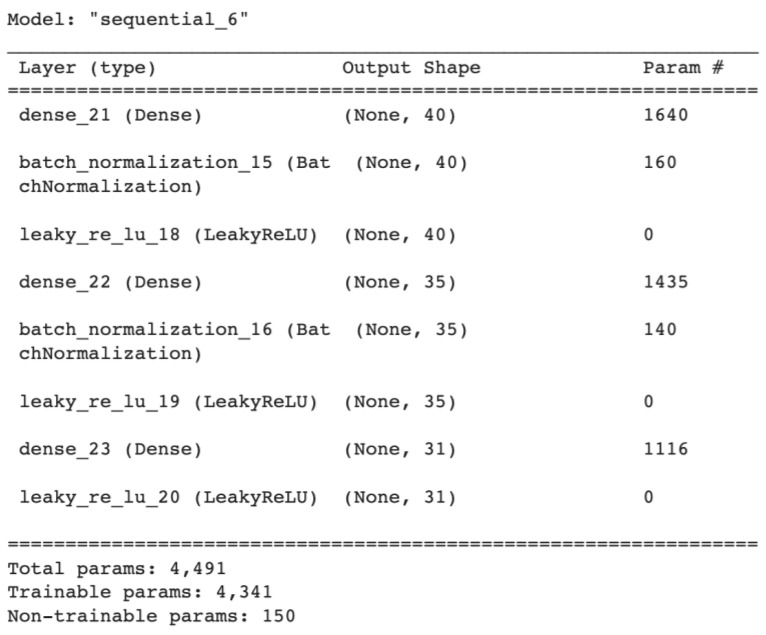
Summary of the generator model.

The model.summary() method offered a graphical representation of the model coupled with the trainable and non-trainable parameters. Figure 22 presents the summary of the generator model, where the Param # represents the number of the parameters for each layer.

#### 2.3.3. The Discriminator Component of the GAN

**Figure 23:**
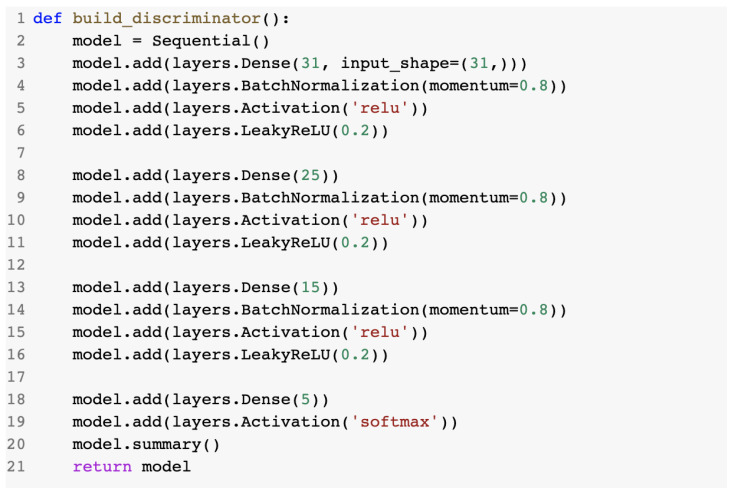
The discriminator model method.

The build_discriminator() method created a sequential model with the same structure as the one from the ANN IDS discussed in Section 2.2.2. The model.summary() method for the discriminator is presented below in Figure 23. We can see the difference in the trainable parameters, as the discriminator model had an input shape of “(None, 31)”, whereas the generator model had an input shape of “(None, 40)”. This was due to the fact that the generator received as input the modified adversarial data entry, whereas the discriminator handled data with the original format.

#### 2.3.4. The Training Algorithm for the GAN

**Figure 24:**
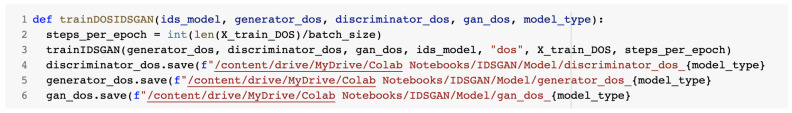
The intermediary training method.

Next, in accordance with the configuration data, the train<attack_type>IDSGAN method was called. This method is intermediary, and it does not include the actual training algorithm but instead performs additional logical operations before calling the actual training method. Figure 24 presents the intermediary training method.

The trainIDSGAN method handled the training of the GAN model. Due to the fact that the GAN model is a relatively new discovery in the machine-learning world, the algorithm for the GAN model had to be written manually. Additional helper methods were used for the fetching of batches of data, creation of the adversarial sample, and retaining of the functional features. The creation of an adversarial sample in the generator was intended to keep the functional features specific to the attack and to change some of the other features.

**Figure 25:**
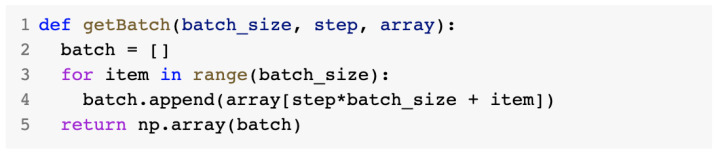
getBatch() method.

The getBatch() method presented in Figure 25 was responsible for returning a batch size for the data used in the training of the model. Batch training was considered to require less memory and it was found to help the model train faster.

**Figure 26:**
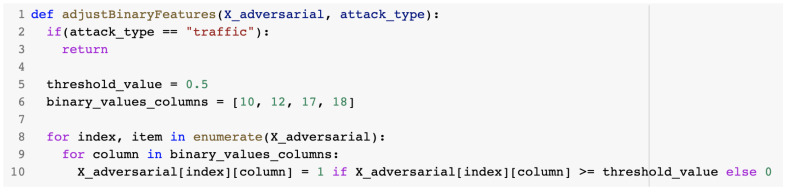
adjustBinaryFeatures() method.

The adjustBinaryFeatures() method described in Figure 26 was used to adjust the binary features from the dataset. Generated binary features were set to the value 0 if the value was below a predefined threshold or to the value 1 if the value was above the threshold.

**Figure 27:**
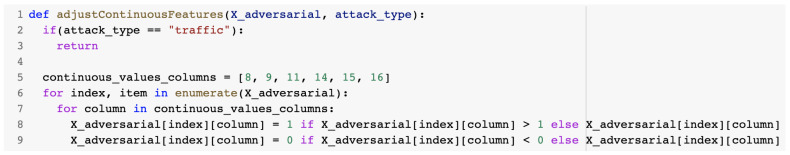
adjustContinuousFeatures() method.

The adjustContinuousFeatures() method described in Figure 27 was used to adjust the continuous features from the dataset. Generated continuous features were set to the value 0 if the generated values were negative and to the value 1 if the generated values were above 1. If they were within the [0, 1] interval, then the generated values were not adjusted. This was required due to the fact that these continuous features were normalized during the data processing step.

**Figure 28:**
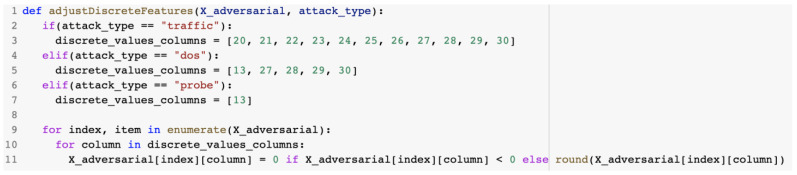
adjustDiscreteFeatures() method.

The adjustDiscreteFeatures() method described in Figure 28 was used to adjust the discrete features from the dataset. Generated discrete features were set to the value 0 if the generated values were negative and to the nearest positive integer if the generated values were above 1.

**Figure 29:**
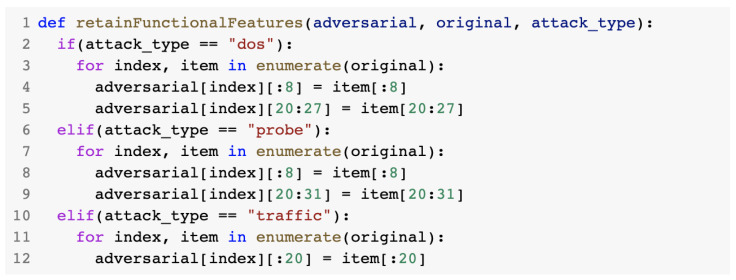
retainFunctionalFeatures() method.

The retainFunctionalFeatures() method was responsible for preserving the functional characteristics of the malicious traffic. It contained three cases: DoS, probe, and traffic (U2R and R2L). If any of the functional features were changed, the traffic was no longer considered to be malicious. For each attack type, a certain set of functional features was intended to remain unchanged in the discriminator process. Figure 29 presents the retainFunctionalFeatures() method.

**Figure 30:**
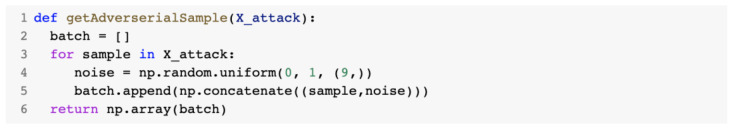
getAdversarialSample() method.

In the getAdversarialSample() method, we appended a random uniformly distributed sample taking values between 0 and 1 and having a shape (9,). This resulted in an adversarial entry that had the shape (40,0). Figure 30 presents the getAdversarialSample() method.

The training of the IDSGAN took place in epochs, with the value being taken from the configuration file described in Section 2.3.1. In order to complete a full epoch, the model was trained in steps that corresponded to the number of batches it took to parse all data. The algorithm was performed with each available batch of data. The steps included in the training are presented below.

Required items:

- Normal and malicious traffic records;
- IDS model;
- Helper methods (discussed previously);
- Initialized GAN components and parameters defined;
- Initial state;
- Discriminator trainable property set to false.

Steps:

1. Retrieve batch of attack data corresponding to current step;
2. Construct adversarial sample;
3. Generate adversarial malicious record for discriminator;
4. Adjust generated features respective to their data type;
5. Retain functional features of original traffic;
6. Retrieve batch of training data corresponding to current step;
7. Classify instances of real and adversarial data using the IDS;
8. Mark discriminator as trainable;
9. Train the discriminator based on the results of the IDS;
10. Update discriminator parameters;
11. Mark discriminator as not trainable;
12. Generate adversarial malicious record for generator;
13. Train generator through the GAN model;
14. Update generator parameters.

**Figure 31:**
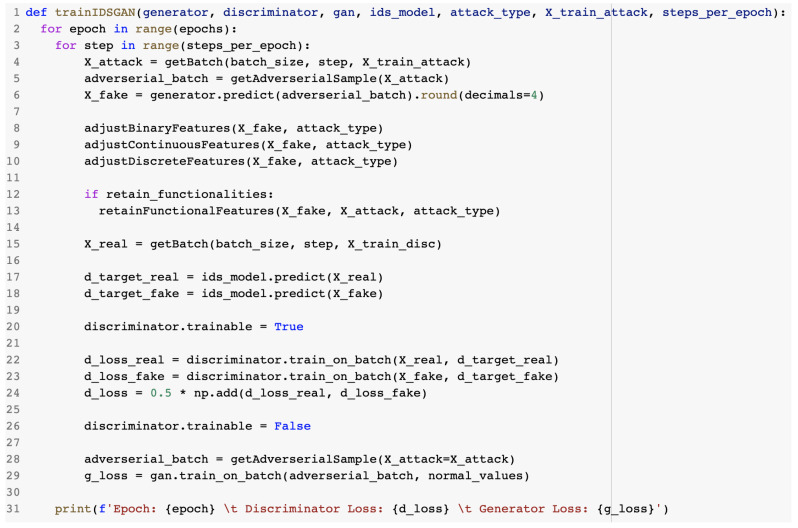
GAN training algorithm.

The training proceeded in alternating periods. The generator was kept constant during the discriminator training phase and, correspondingly, the discriminator was kept constant during the generator training phase. Figure 31 presents the GAN training algorithm. The discriminator loss d_loss is computed as the median of the two matrices, d_loss_real and d_loss_fake.

#### 2.3.5. Testing and Tuning the IDS by Launching the Adversarial Attack

The testing of the GAN adversarial attack was undertaken in the GAN Adversarial Attack.ipynb notebook. This step was dedicated to testing the attacking capabilities of our implementation. The purpose of this attack was to make the IDS perceive generated adversarial malicious traffic as normal traffic and, therefore, to access the system. In this way, we could both test the performance of the IDS and also, by changing the saved model of the IDS, tune that component.

**Figure 32:**
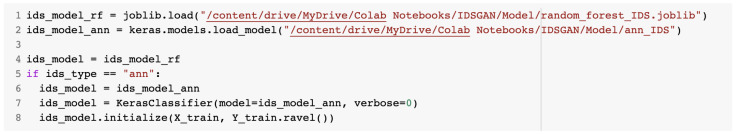
Loading of the IDS to be tested and tuned by the GAN.

This step also made use of the configuration file to specify the scenario we wanted to verify. For this, we loaded the testing dataset and selected the IDS according to the configuration file. The source code that was used is presented in Figure 32.

**Figure 33:**
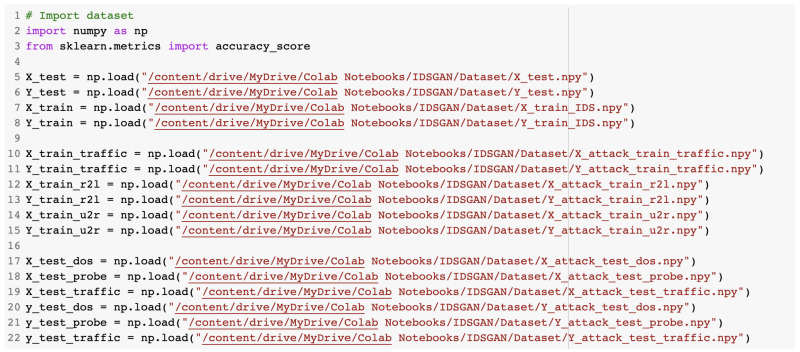
Loading of the testing dataset.

After this, the testing dataset was loaded as presented in Figure 33. On the first line, # represents a comment in the source code.

Next, the generator models were loaded. They were created and trained as described in Section 2.2.2. In order to estimate the GAN performance and the IDS performance in the presence of the GAN, we verified the IDS by predicting the real attack data, and the results are described in Section 2.2.3. Then, the same predictions were computed using the adversarial dataset. This helped us obtain a clear view of how the IDS was affected by the adversarial traffic.

**Figure 34:**
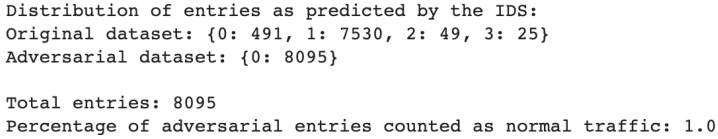
IDS prediction of adversarial entries.

The percentage of adversarial entries perceived as normal traffic was computed in order to calculate the effectiveness of the adversarial attack and is presented in Figure 34.

**Figure 35:**
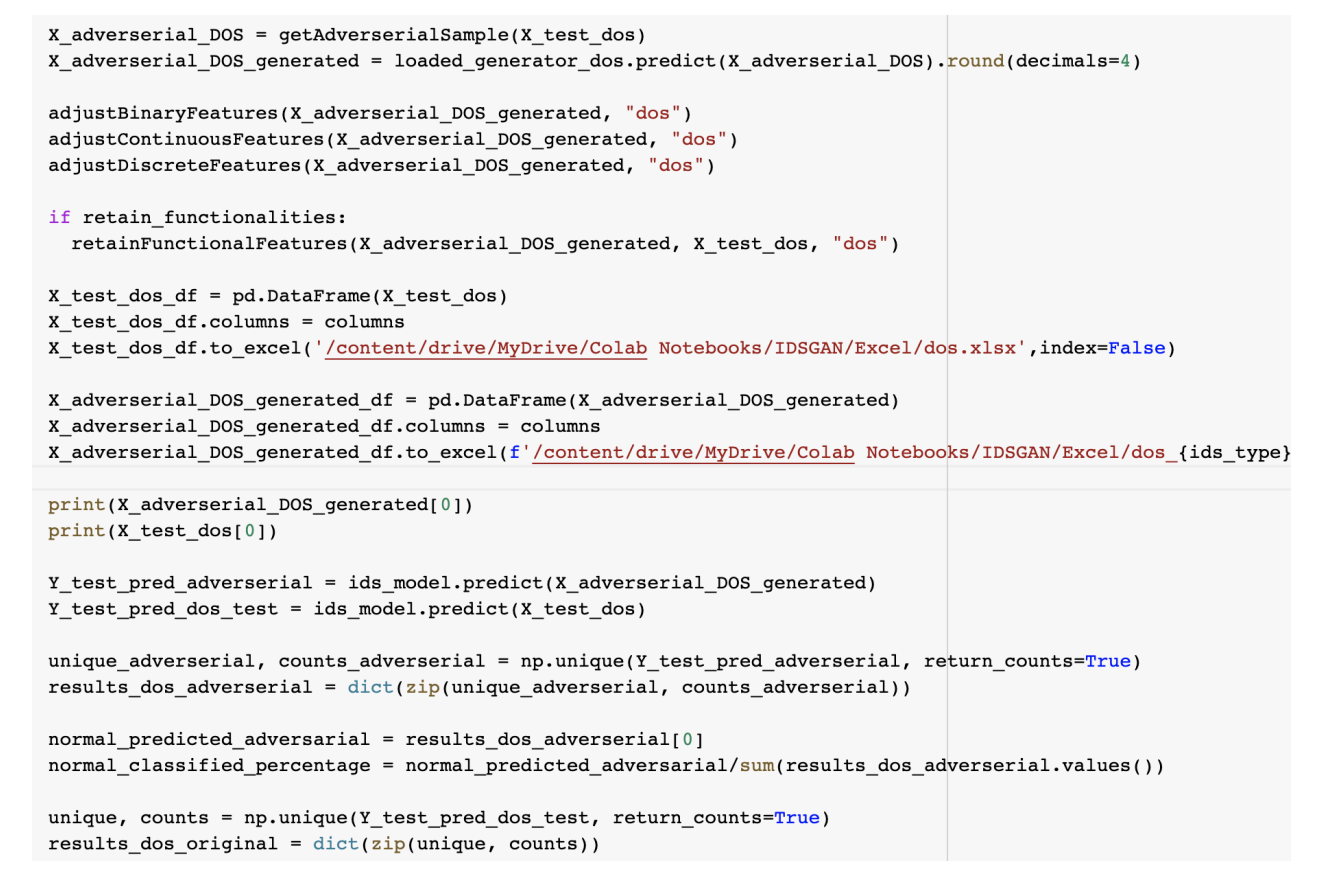
Computing the results of the adversarial attack classification.

The source code used to compute the results of the adversarial attack classification is presented in Figure 35.

In addition to these measurements, the detection rate and the evasion increase rate were calculated. The original detection rate and the adversarial detection rate were the detection rate compared to the original malicious traffic records and to the adversarial malicious traffic records, respectively. In addition, the evasion increase rate (EIR) was the rate of the increase in undetected adversarial malicious traffic by the IDS, measuring the attack evasion efficiency of the GAN [13]. Equations (4) and (5) present these metrics. Lower values for the EIR indicate better performance for the IDS in the presence of adversarial traffic.

(4) $$\mathrm{DR}=\frac{Number\;of\;attacks\;detected}{Total\;number\;of\;attacks}$$

(5) $$\mathrm{EIR}=1-\frac{Detection\;rate\;in\;the\;case\;of\;adversarial\;attacks}{Original\;detection\;rate}$$

**Figure 36:**
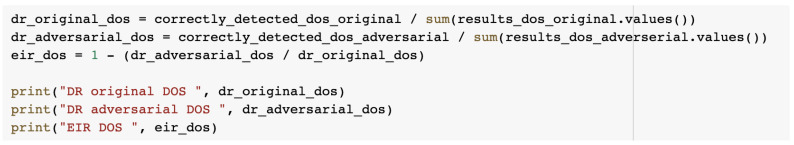

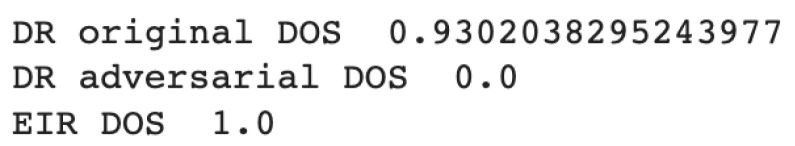
DR and EIR computation.

The computation of the DR and EIR parameters is presented in Figure 36.

**Figure 37:**
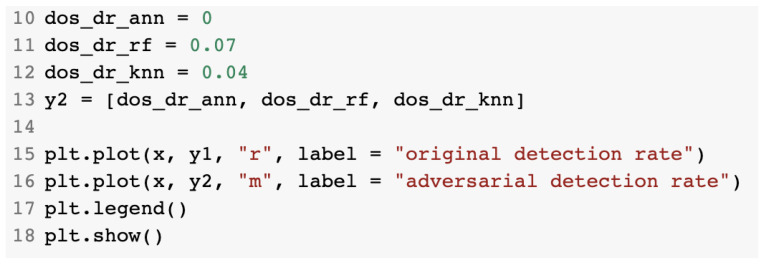
Plotting of generative model results.

Finally, we displayed the overall results of the constructed generative models using the matplotlib.pyplot plotting library using the source code presented in Figure 37.

## 3. Results

**Figure 38:**
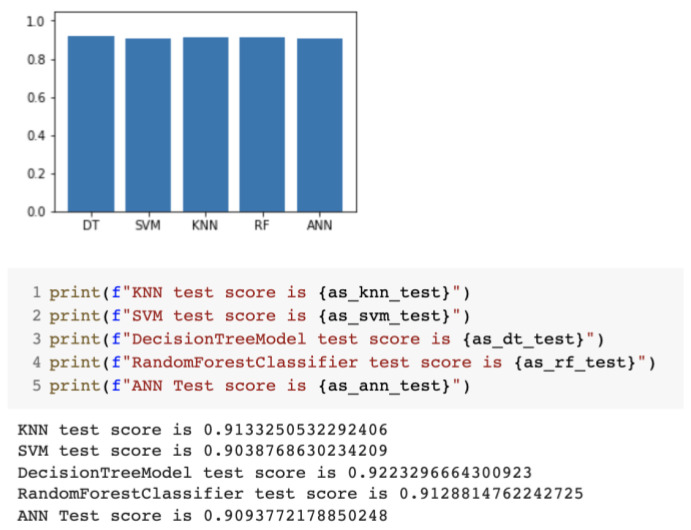
Performance of the IDS models.

First, the performance of the IDS models with the NSL-KDD dataset was assessed. Then, the performance of the ID models was evaluated in the presence of the GAN traffic. Figure 38 shows that the IDS models performed very well, all exhibiting accuracy close to or over 0.9. However, only the ANN, random forest, and K-nearest neighbor classifiers were used in the testing and tuning with the GAN sections.

For each model, the confusion matrix was created and the precision, recall, and F1 factor were computed.

Next, the performance of the tested models with the GAN-generated traffic is presented. We tested the algorithm performance with adversarial traffic in two situations for each attack category and compared it with the initial performance with the original NSL-KDD dataset.

### 3.1. Asessing the Performance in the Case of Probe Attack Traffic

#### 3.1.1. ANN IDS Performance in the Case of Generated Adversarial Probe Traffic

For the case of probe traffic detection, we tested and compared the performance with the original NSL-KDD database (which contained 2157 probe records) against two configurations of the ANN IDS that received generated adversarial traffic. Both the configurations that were tested in this case for the discriminator used the ANN that was previously implemented as the IDS with the NL-KDD dataset as input.

Table 11 presents the simulation parameters used with the ANN for detecting probe attacks.

Table 12 presents the probe attack detection in the case of the ANN. The first column specifies the predicted attack category. In an ideal situation, only probe traffic should be detected. The second column specifies the results for the initial ANN IDS implementation that was trained using the original NSL-KDD dataset. The third and the fourth columns specify the results obtained with the GAN setup when the discriminator implemented with the ANN was tested with the generated adversarial traffic. The results in column three were obtained using the ADAM optimizer, whereas the results in column four were obtained using the stochastic gradient descent (SGD) optimizer.

As the total number of probe records was 2157, the detection rate (DR) for the initial IDS test with the original NSL-KDD dataset was 83.8%. For the ANN using the ADAM discriminator, the percentage of adversarial entries classified as normal data was 94.6%. The detection rate for the adversarial probe data was 0. The evasion increase rate was 1.0. For the ANN that used SGD, the percentage of adversarial entries classified as normal data was 73.75%. These results are presented in Table 13.

Table 12 and Table 13 show the advantages of using adversarial training in the development of IDS systems. Even if the IDS performance decreased significantly after careful training of the GAN generator with the IDS, the detection performance could be improved and offers benefits over training using information from the original dataset.

#### 3.1.2. Random Forest IDS Performance in the Case of Generated Adversarial Probe Traffic

Table 14 presents the results obtained with the use of the random forest model for the IDS.

Table 15 presents the performance of the random forest model.

Table 14 and Table 15 highlight the better performance of the random forest algorithm compared to the previous ANN algorithm.

#### 3.1.3. kNN IDS Performance in the Case of Generated Adversarial Probe Traffic

Table 16 and Table 17 show the performance of the kNN algorithm in the case of adversarial traffic. Table 17 presents the performance of the kNN algorithm.

As shown in Table 17, in the case of kNN, the ADAM optimizer performed better than SGD.

### 3.2. Asessing the Performance in the Case of DoS Attack Traffic

The results with DoS traffic are displayed for the ANN, random forest, and kNN IDSs.

#### 3.2.1. ANN IDS Performance in the Case of Generated Adversarial DoS Traffic

Table 18 presents the DoS attack detection in the case of ANN as discriminator.

Table 19 presents the performance of the ANN.

As shown in Table 19, with the ANN, both the ADAM and the SGD optimizers performed poorly.

#### 3.2.2. Random Forest IDS Performance in the Case of Generated Adversarial DoS Traffic

Table 20 presents the values measured in the case of the Random Forest discriminator in the presence of generated adversarial DoS traffic.

Table 21 presents the performance of the Random Forest algorithm.

As shown in Table 21, with the random forest algorithm, the SGD optimizer performed better than the ADAM optimizer.

#### 3.2.3. kNN IDS Performance in the Case of Generated Adversarial DoS Traffic

Table 22 presents the DoS attack detection in the case of kNN used for the discriminator.

Table 23 presents the performance of the kNN algorithm.

As shown in Table 23, with kNN, the SGD optimizer performed significantly better than the ADAM optimizer.

## 4. Discussion

In this paper, we investigated the effect of adversarial data generated using a GAN on an IDS implementing machine-learning algorithms. First, we implemented the IDS and demonstrated its performance with each algorithm. Then, we implemented a GAN where the discriminator used one of the previously tested algorithms and the generator used the same algorithm and tried to maximize the loss of the discriminator. We compared the original performance (when tested with the NSL-KDD dataset) with the performance in the presence of the adversarial traffic. The results described in Section 3 are summarized in Section 4.1 for the case of probe traffic detection and Section 4.2 for the case of DoS traffic detection.

### 4.1. Comparison between ML Methods in the Case of Probe Traffic

In Table 24, we display the results we obtained using the ANN, random forest, and kNN classifiers for the case of KDD traffic compared to the results obtained in the presence of the generated adversarial probe traffic.

**Figure 39:**
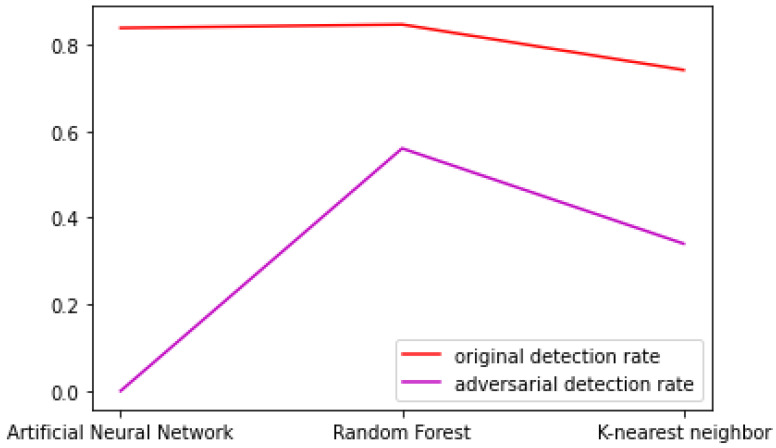
Comparison between probe original and adversarial detection rates using different IDS models.

From Table 24, it can be seen that the generative model performed very well in evasion when the black-box IDS used was based on an artificial neural network, but the IDS was not capable of correctly classifying the adversarial traffic. When the random forest algorithm was used as a classifier for the IDS, the detection and evasion rates were worse than the neural network, but the traffic still succeeded, to a small extent, in fooling the IDS. Therefore, the random forest algorithm seemed to be a more appropriate solution for the implementation of an IDS in the presence of a GAN-generated traffic attack. Figure 39 presents a comparison of the original (using KDD training data) detection rate and the adversarial detection rate in the case of probe traffic.

### 4.2. Comparison between ML Methods in the Case of DoS Traffic

It the case of DoS traffic Table 25 presents the comparative performances of DR and EIR for the three ML method studied (ANN, Random Forest and kNN) in the presence of the original NSL-KDD traffic and the adversarial traffic.

It can be seen that the generative model performed very well in the process of evasion when the IDS used was based on an artificial neural network but, similarly to the probe traffic, the IDS was not capable of correctly classifying the adversarial traffic.

**Figure 40:**
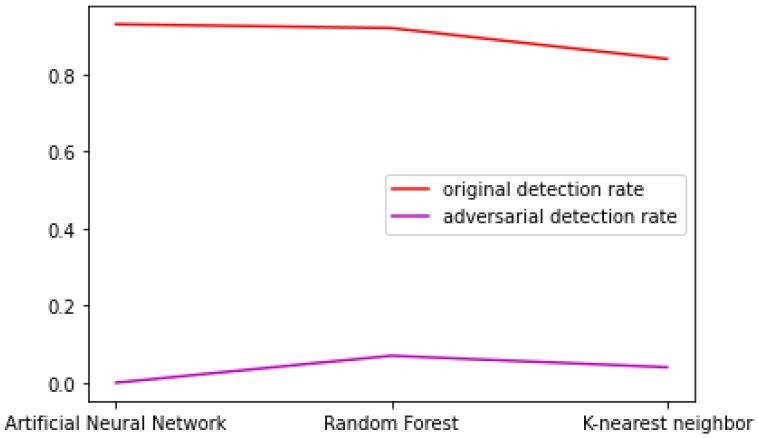
Comparison of DoS original and adversarial detection rates using different IDS models.

When the random forest algorithm was used as a classifier for the IDS, the detection and evasion rates were worse than the neural network but still very good. This could be attributable to the fact that the DoS dataset was larger than the probe one, and the model succeeded in learning the pattern in the traffic records better than in the previous case. Figure 40 presents a comparison between the different IDS models in the case of DoS traffic. It is clear that, in the case of DoS, the performance of the IDS must be improved because the adversarial detection rate was very low for each method. One possible approach to increase the detection rate in the case of adversarial traffic is to include adversarial samples in the training dataset or to use additional datasets. A third option would be to implement a honeypot [7] with feature extraction capabilities. This would collect new attack traffic with the same functional features as in the dataset. The new records would be beneficial for training the models for U2R and R2L.

## 5. Conclusions and Future Work

In this paper, we implemented an end-to-end flow for creating adversarial generated network traffic and testing its malicious potential using the generative adversarial network framework. The purpose of the generated network traffic was to evade machine-learning intrusion detection systems while keeping the functional features of its respective attacking types intact. In order to achieve this, we proceeded through various steps: acquisition of the NSL-KDD dataset, preprocessing of the training dataset, creation of multiple intrusion detection systems using diverse machine-learning algorithms, and implementation of the generative adversarial network model. Finally, by connecting all of these building blocks, we generated adversarial malicious traffic and successfully tested its attacking and evading capabilities.

For the technical implementation of these steps, we used the Google Colab Jupyter notebook service, in which we made use of data science tools, such as the numpy and pandas libraries, and machine-learning-oriented frameworks, such as TensorFlow and Keras, to create the actual machine-learning models for the IDS and the GAN. The programming language used for coding all of these items was Python, which is the perfect tool for both simple and complex data manipulation.

In order to train and generate the GAN model, we devised multiple IDS machine-learning models using diverse machine-learning algorithms, such as the random forest algorithm and an artificial neural network, and we managed to obtain stable classifiers that we used as a foundation for the training process with the GAN model. Out of all the attacking types, DoS resulted in the best performance in terms of detection by the IDS, which was expected, as it had the most numerous traffic entries with the exception of the normal traffic type. In order to devise a configurable method of training the GAN model, we used a JSON configuration file with which we managed to easily tweak the hyperparameters of the GAN training. Furthermore, we managed to write a functional algorithm representing the training requirements of the GAN model architecture and obtain functional generative models, which were used later to generate the adversarial network traffic.

The generated adversarial malicious traffic was used to test the detection capabilities of the IDS models. For this, we used three attack types: DoS, probe, and traffic (R2U and U2R). Among these three types, the DoS and probe types were very successful in evading the detection capabilities of the IDS, the DoS-generated instances even being close to evading them completely, while retaining their functional features. It can be concluded that our generative model succeeded in creating network traffic instances that were successful in evading the intrusion detection system while retaining the functional features of their attacking types. We also demonstrated that the discriminator and the generator components from a GAN architecture can be used in order to improve the performance of a machine-learning IDS by feeding the discriminator with the output from the generator.

For three attack categories (probe, U2R, and R2L), the detection performance was very low due to the low number of records available in the training dataset. For future work, we intend to improve the detection performance for these classes by using the generator to generate new records for the training dataset while retaining the functional features.

Another direction for future work will be to test the system with real traffic by extracting relevant features from live packets and feeding them to the machine-learning IDS system. This approach will enable measurement of the classification delay, which is critical for IDS systems.

## Figures and Tables

**Table 1:** Performance analysis of the kNN IDS.

Predicted Attack Category	Precision	Recall	F1 Score
Normal	0.92	0.98	0.95
DoS	0.95	0.93	0.94
Probe	0.92	0.67	0.77
R2L	0.52	0.41	0.46
U2R	0	0	0

**Table 2:** Confusion matrix for the kNN IDS.

Predicted Attack Category	Normal	DoS	Probe	R2L	U2R
Normal	**97.91% (32,751)**	0.94% (317)	0.76% (257)	0.36% (122)	0
DoS	6.30% (1456)	**93.26% (21,553)**	0.37% (87)	0.06% (14)	0
Probe	18.72% (1100)	13.58% (798)	**66.58% (3912)**	1.10% (65)	0
R2L	57.57% (304)	0	1.70% (9)	**40.72% (215)**	0
U2R	96.23% (26)	3.7% (1)	0	0	**0**

**Table 3:** Performance analysis of the decision tree IDS.

Predicted Attack Category	Precision	Recall	F1 Score
Normal	0.98	0.99	0.99
DoS	1.00	0.99	1.00
Probe	0.96	0.95	0.95
R2L	0.94	0.78	0.85
U2R	1.00	0.22	0.36

**Table 4:** Confusion matrix for the decision tree IDS.

Predicted Attack Category	Normal	DoS	Probe	R2L	U2R
Normal	**99.12% (33,152)**	0.14% (48)	0.66% (221)	0.07% (26)	0
DoS	0.49% (113)	**99.4% (22,972)**	0.1% (25)	0	0
Probe	4.87% (286)	0.03% (2)	**95.08% (5586)**	0.01% (1)	0
R2L	22.35% (118)	0	0	**77.65% (528)**	0
U2R	77.78% (21)	3.7% (1)	0	0	**22.22% (6)**

**Table 5:** Performance analysis of the random forest IDS.

Predicted Attack Category	Precision	Recall	F1 Score
Normal	1.00	1.00	1.00
DoS	1.00	1.00	1.00
Probe	1.00	1.00	1.00
R2L	1.00	0.99	0.99
U2R	1.00	0.89	0.94

**Table 6:** Confusion matrix for the random forest IDS.

Predicted Attack Category	Normal	DoS	Probe	R2L	U2R
Normal	**99.99% (33,443)**	0.002% (1)	0.008% (3)	0	0
DoS	0.02% (6)	**99.97% (23** **,103)**	0.004% (1)	0	0
Probe	0.10% (6)	0	**99.89% (5869)**	0	0
R2L	1.13% (6)	0	0	**98.86% (522)**	0
U2R	11.11%(3)	0	0	0	**88.88% (24)**

**Table 7:** Performance analysis of the SVM IDS.

Predicted Attack Category	Precision	Recall	F1 Score
Normal	0.86	0.99	0.92
DoS	0.96	0.92	0.94
Probe	0.93	0.37	0.53
R2L	0.00	0.00	0.00
U2R	0.00	0.00	0.00

**Table 8:** Confusion matrix for the SVM IDS.

Predicted Attack Category	Normal	DoS	Probe	R2L	U2R
Normal	**99.29% (33,210)**	0.52% (175)	0.18% (62)	0	0
DoS	7.75% (1791)	**91.8% (21,220)**	0.43% (99)	0	0
Probe	49.58% (2913)	13.65% (802)	**36.77% (2160)**	0	0
R2L	99.24% (524)	0	0.76% (4)	**0**	0
U2R	96.3% (26)	3.7% (1)	0	0	**0**

**Table 9:** Performance analysis of the ANN IDS.

Predicted Attack Category	Precision	Recall	F1 Score
Normal	0.99	0.99	0.99
DoS	1.00	1.00	1.00
Probe	0.98	0.96	0.97
R2L	0.81	0.79	0.80
U2R	1.00	0.56	0.71

**Table 10:** Confusion matrix for the ANN IDS.

Predicted Attack Category	Normal	DoS	Probe	R2L	U2R
Normal	**99.17% (33,169)**	0.19% (63)	0.36% (122)	0.28% (93)	0
DoS	0.06% (13)	**99.92% (23,093)**	0.01% (3)	0.004% (1)	0
Probe	3.52% (207)	0.1% (6)	**96.36% (5661)**	0.02% (1)	0
R2L	20.83% (110)	0	0	**79.17% (418)**	0
U2R	25.92% (7)	3.7% (1)	3.7% (1)	11.11% (3)	**55.56% (27)**

**Table 11:** Simulation parameters used with the ANN with generated adversarial probe traffic.

Parameter	Value
Algorithm	ANN
Epochs	32
Batch size	32
Retain functional parameters	Yes
Optimizer	ADAM or SGD

**Table 12:** Probe attack detection with the ANN used for the discriminator.

Predicted Attack Category	Initial IDS Testing	ANN, ADAM	ANN, SGD
Normal	265	2041	1591
DoS	84	22	258
**Probe**	**1808**	**0**	**280**
R2L	0	94	28
U2R	0	0	0

**Table 13:** Performance of the ANN.

Parameters	Initial IDS Testing	ANN, ADAM	ANN, SGD
Detection rate (DR)	0.838	0	0.1298
Evasion increase rate (EIR)	N/A	1.0	0.845
Percentage of adversarial entries classified as normal data (%)	N/A	94.6	73.75

**Table 14:** Probe attack detection with the use of the random forest model for the discriminator.

Predicted Attack Category	Initial IDS Testing	RF, ADAM	RF, SGD
Normal	285	717	1591
DoS	45	1	258
**Probe**	**1827**	**1430**	**280**
R2L	0	9	28
U2R	0	0	0

**Table 15:** Performance of the random forest model.

Parameters	Initial IDS Testing	RF, ADAM	RF, SGD
Detection rate (DR)	84.6	0.66	0.56
Evasion increase rate (EIR)	N/A	0.21	0.32
Percentage of adversarial entries classified as normal data (%)	N/A	33.2	42

**Table 16:** Probe attack detection with kNN used for the discriminator.

Predicted Attack Category	Initial IDS Testing	kNN, ADAM	kNN, SGD
Normal	399	722	1427
DoS	156	15	10
**Probe**	**1600**	**1402**	**713**
R2L	2	18	7
U2R	0	0	0

**Table 17:** Performance of kNN algorithm.

Parameters	Initial IDS Testing	kNN, ADAM	kNN, SGD
Detection rate (DR)	0.74	0.64	0.33
Evasion increase rate (EIR)	N/A	0.123	0.55
Percentage of adversarial entries classified as normal data (%)	N/A	33.5	66.2

**Table 18:** DoS attack detection with the use of the ANN for the discriminator.

Predicted Attack Category	Initial IDS Testing	ANN, ADAM	ANN, SGD
Normal	500	1690	8095
**DoS**	**7557**	**884**	**0**
Probe	38	0	0
R2L	0	5419	0
U2R	0	102	0

**Table 19:** Performance with DoS when using the ANN IDS.

Parameters	Initial IDS Testing	ANN, ADAM	ANN, SGD
Detection rate (DR)	0.9335	0.0	0.0
Evasion increase rate (EIR)	N/A	0.99	1.0
Percentage of adversarial entries classified as normal data (%)	N/A	99.9	100

**Table 20:** DoS attack detection with the use of the random forest algorithm for the discriminator.

Predicted Attack Category	Initial IDS Testing	RF, ADAM	RF, SGD
Normal	558	5638	3420
**DoS**	**7536**	**591**	**4540**
Probe	1	1886	0
R2L	0	0	135
U2R	0	0	0

**Table 21:** Performance with DoS when using the random forest IDS.

Parameters	Initial IDS Testing	RF, ADAM	RF, SGD
Detection rate (DR)	0.921	0.07	0.56
Evasion increase rate (EIR)	N/A	0.92	0.39
Percentage of adversarial entries classified as normal data (%)	N/A	66.9	42.2

**Table 22:** DoS attack detection with the use of kNN for the discriminator.

Predicted Attack Category	Initial IDS Testing	kNN, ADAM	kNN, SGD
Normal	887	7726	4685
**DoS**	**7177**	**324**	**2765**
Probe	30	42	504
R2L	1	3	141
U2R	0	0	0

**Table 23:** Performance with DoS when using the kNN IDS.

Parameters	Initial IDS Testing	kNN, ADAM	kNN, SGD
Detection rate (DR)	0.8865	0.04	0.34
Evasion increase rate (EIR)	N/A	0.95	0.61
Percentage of adversarial entries classified as normal data (%)	N/A	95.4	57.9

**Table 24:** IDS rates for KDD and generated adversarial probe traffic.

ML Method Used	DR, KDD Traffic	ADAM DR	ADAM EIR	SGD DR	SGD EIR
ANN	0.838	0.0	1.0	0.1298	0.845
Random forest	0.846	0.66	0.21	0.56	0.32
kNN	0.741	0.64	0.123	0.33	0.55

**Table 25:** IDS rates for KDD and generated adversarial DoS traffic.

ML Method Used	DR, KDD Traffic	ADAM DR	ADAM EIR	SGD DR	SGD EIR
ANN	0.93	0.0	0.99	0.0	1.0
Random Forest	0.92	0.07	0.92	0.56	0.39
kNN	0.84	0.04	0.95	0.34	0.61

## Data Availability

The data that support the findings of this study are available from the corresponding author (D.Z.) upon reasonable request.
